# Comparison of Different Machine Learning Models for Predicting Long-Term Overall Survival in Non-metastatic Colorectal Cancers

**DOI:** 10.7759/cureus.75713

**Published:** 2024-12-14

**Authors:** Fahriye Tugba Kos, Songul Cecen Kaynak, Selin Aktürk Esen, Hilal Arslan, Dogan Uncu

**Affiliations:** 1 Department of Medical Oncology, Ankara Bilkent City Hospital, Ankara, TUR; 2 Department of Software Engineering, Faculty of Engineering and Natural Sciences, Ankara Yıldırım Beyazıt University, Ankara, TUR

**Keywords:** artificial intelligence (ai), (crc) colorectal carcinoma, machine learning (ml), non-metastatic, survival

## Abstract

Introduction: In recent years, machine learning (ML) methods have gained significant popularity among medical researchers interested in cancer. We aimed to test different (ML) models to predict both overall survival and survival at specific time points in patients with non-metastatic colorectal cancer (CRC).

Methods: The clinicopathological and treatment data of non-metastatic CRC patients with more than 10 years of follow-up at a single center were retrospectively reviewed. 1, 2, 3, 5, and 10-year survival rates for all patients and stages I-III were statistically calculated using the Kaplan-Meier method. Five distinct machine-learning algorithms were employed to develop predictive models for patient survival at five designated time points.

Results: A total of 498 patients were included in the study. The decision tree model had the highest area under the curve (AUC) for 1-year survival prediction (0.89). The ensemble model had the highest AUC for predicting 2-year, 3-year, and 5-year survival predictions (0.86, 0.92, and 0.89, respectively), while the support vector machine model had the highest AUC (0.84) for predicting 10-year survival. When considering the stages separately and assessing survival for the designated time intervals, the accuracy of all five models was found to be similar, ranging around 70% or higher.

Conclusion: ML models can predict short- and long-term survival in patients with CRC, both for the overall patient population and when stratified by stage.

## Introduction

Colorectal cancer (CRC) is the third most commonly diagnosed cancer in men and the second most commonly diagnosed cancer in women, and, according to the World Health Organization’s GLOBOCAN database [1]. It is estimated that 1.93 million new cases of CRC will be diagnosed and 0.94 million deaths from CRC will occur worldwide in 2020, accounting for 10% of the global cancer incidence (19.29 million total new cases) and 9.4% of all cancer deaths (9.96 million total deaths) [2]. Cases of CRC diagnosed in the early stages may offer more aggressive treatment options and better chances of survival, while survival rates are often lower in cases diagnosed at later stages. Stage at diagnosis is the most important predictor of survival, with 5-year relative survival rates ranging from 91% for localized disease to 14% for distant disease [3].

Contemporary research in early-stage (stage I to III) colon cancer focuses on minimally invasive surgical techniques, strategies to limit treatment-related toxicities, precise patient selection for adjuvant therapy, the use of molecular and clinicopathological information to personalize treatment, and the search for new treatments by taking advantage of evolving knowledge about the tumor [4]. The primary treatment for early-stage colon cancer is surgery. Adjuvant therapy is recommended in selected patients with stage II disease. In stage III patients, the 5-year disease-free survival (DFS) rate of 45-50% with surgery alone can be increased to 67% with adjuvant chemotherapy [4]. Similarly, research is ongoing to improve survival in rectal carcinoma. Similarly, research is ongoing to improve survival in rectum carcinoma. In light of the data on multimodal treatments, total neoadjuvant treatment options with short or long-course chemoradiotherapy are employed in patients with T3/T4 or node-positive or positive circumferential radial margin [5].

While the data obtained from clinical studies continue to elucidate the unknowns in cancer treatment, the rapid development of technology has brought about the increased integration of artificial intelligence (AI) into medical studies. AI can be classified into distinct subfields: machine learning (ML) and deep learning (DL). Recent advances in ML techniques have demonstrated high predictive accuracy and are being widely used to diagnose and predict a range of diseases and medical conditions [6,7]. In recent years, ML methods have gained significant popularity among medical researchers interested in cancer [8]. ML is a data-centered approach that combines various risk factors to form predictive algorithms. ML algorithms can improve their performance through experimentation and iteration. Algorithms such as decision trees [9,10] and support vector machines (SVMs) [11,12] have been used to identify key patient features and model disease progression using comprehensive health data.

The literature has also shown that ML models using factors such as gender, age, and blood cell count can effectively detect early colorectal cancer [13]. Predicting survival is very difficult in malignant disease, but it is important for treatment planning and patient management [14]. The accuracy of cancer prognosis prediction will greatly benefit the clinical management of cancer patients. The application of AI to predict cancer prognosis prediction in various cancers has significantly improved the performance of cancer prognosis prediction [15].

Although the number of studies using artificial intelligence in oncology is increasing every day, there is still a lack of studies using machine learning models to predict survival in early-stage colorectal cancer using clinical data. It is well known that survival decreases as the stage of colorectal cancer progresses. In oncology, it is important to determine the proportion of patients who are alive at certain time points in order to track improvements in survival following interventions performed on patients. Given this information, our study aimed to test different machine-learning models to determine both overall survival and survival at specific time intervals in early-stage colorectal cancer patients in our clinic.

## Materials and methods

Selection of patients

Patients with CRC from the institutional database of Ankara Bilkent City Hospital (formerly known as Ankara Numune Education and Research Hospital) in the Department of Medical Oncology were retrospectively reviewed. In order to assess 10-year and longer periods of overall survival, patients who were diagnosed before January 2014 were included. Patients with metastatic disease were excluded. The clinicopathological characteristics of the patients and their treatment data were evaluated using the hospital's automation and archiving system. Patients' age at diagnosis, gender, tumor location, tumor stage, pathological features, the number of chemotherapy cycles and regimens received in neoadjuvant or adjuvant treatment, chemotherapy response, and whether recurrence occurred were recorded. These characteristics were chosen because they are generally known from previous research to be important factors influencing survival. The tumor-node-metastasis staging system of the American Joint Committee on Cancer (7th edition) was used for staging. Patients with incomplete data were excluded. The primary outcomes were the machine learning model's predictions of 1, 2, 3, 5, and 10-year overall survival for the study population and each stage (I-III). The Ankara Bilkent City Hospital Medicine and Health Sciences Research Ethics Committee gave approval for this study (TABED 1-24-143).

Statistical analysis

Overall survival was determined by time from the diagnosis to the last visit or death. Patients' survival data were updated in February 2024. SPSS version 24.0 (IBM Corp., Chicago) package program was used for statistical analysis. A p-value <0.05 was considered statistically significant. Continuous variables are expressed as median (range). Categorical variables were expressed as numbers and percentages. The Chi-squared test or Fisher's exact test, as appropriate, was used for correlations between clinicopathological parameters. Survival analyses were calculated using the Kaplan-Meier method.

Machine learning methods

In this study, decision tree (DT), support vector machine (SVM), K-nearest neighbor (KNN), ensemble classifier, and neural network (NN) supervised ML classification methods were applied. All computations were performed on a computer equipped with an 11th generation Intel®️Core™️ i7-1165G7 processor running at 2.80 GHz with 16 GB of RAM, using MATLAB R2023b Update 7 software.

We used ANOVA, Kruskall-Wallis, and Chi-square methods to present the importance of the features. They give similar results, so we have only given the results of the ANOVA method. To evaluate the performance of the ML method, the k-fold cross-validation technique is used, where k is conventionally set at 10. In this approach, the data set is randomly divided into ten subsets (i.e., folds) of equal size. Each time, one of the ten subsets is used for testing, and the remaining nine subsets are used for training. This process is repeated ten times so that each subset is used exactly once as a test dataset. We presented the AUC scores, accuracy, F-measure, precision, and recall results of the ML methods to evaluate and compare the results.

A decision tree is a type of ML model that helps identify patterns in data. It works by taking a set of input values and then splitting them into different branches depending on what the tree believes is the best decision for the available data [16]. Each decision the tree makes can be seen as a step in determining the best possible option. The final result of a DT is usually a specific set of output values, reflecting the probability that each input value corresponds to one of the predetermined output values. This model can be used for different tasks, such as pattern recognition, prediction, and classification. This study uses three DT ML modeling methods: fine tree, medium tree, and coarse tree.

SVM is a commonly used method for classification problems because it can handle both linear and non-linear classification tasks. It aims to create a decision boundary between two classes, known as a hyperplane, which allows labels to be predicted from one or more feature vectors. This hyperplane is oriented to maximize the distance between the closest data points of opposite classes. These closest points are called support vectors. If the data is not linearly separable, kernel functions are used to transform the data into a higher dimensional space to allow linear separation [17]. This study applies six SVM methods: linear SVM, quadratic SVM, cubic SVM, fine Gaussian SVM, medium SVM, and coarse SVM.

A neural network is an ML model that makes decisions in a manner similar to that of the human brain [18]. It uses processes that mimic the way biological neurons work together to identify the p. Each NN consists of layers of nodes or artificial neurons, an input layer, single or multiple hidden layers, and an output layer. Each node is connected to others and is associated with a particular weight and threshold. When the output of an individual node c exceeds the specified threshold value, then the node is activated, and the data is transmitted to the next layer in the network. Neural networks learn and improve their accuracy over time by training on data. This study uses five NN ML modeling methods: narrow NN, medium NN, wide NN, bilayered NN, and trilayer NN.

K-Nearest Neighbor (KNN) is a supervised ML method used to classify unlabelled data into the most appropriate class based on their proximity to other data points in the training set. The algorithm classifies new data points by comparing their features with the labeled data points in the training set [19]. Since KNN does not require training data points for model generation, it is considered a lazy algorithm that uses all training data during the testing phase. This study uses the following KNNs: fine KNN, medium KNN, coarse KNN, coarse KNN, Cuc KNN, and weighted KNN.

 Ensemble classifiers combine multiple models to improve the ML results and overall predictive performance compared to a single model [20]. The ensemble classifiers used in this study are boosted trees, bagged trees, subspace discrimant, subspace KNN, and rusboosted trees.

## Results

The records of 1706 CRC patients registered in our clinic's archive were scanned. Of these, 498 patients who were non-metastatic and had all parameters available were included in the study. Localization of the tumor in patients was 224 (45.0%) in rectum, 219 (44.0%) colon, and 55 (11.0%) rectosigmoid. Sixty-three (42.9%) patients were stage I, 237 (46.6%) stage II, and 198 (39.8%) stage III. Patients received neoadjuvant and adjuvant fluorouracil-based treatments. Neoadjuvant radiotherapy was administered simultaneously with the infusional FUFA regimen. During the adjuvant period, FUFA/capecitabine (50.8%) and FOLFOX/XELOX (49.2%) regimens were preferred. The clinicopathological and treatment data of the patients are summarized in Table 1.

**Table 1:** General characteristics of the patients. CT: chemotherapy, CRT: chemoradiotherapy. The data has been represented as n(%) and Median (range).

General characteristics of the patients	Variables	N, %
Median age (range)		59 (22-95)
Gender, n(%)	Female	197 (39.6)
	Male	301 (60.4)
Tumor localization, n(%)	Colon	219 (44.0)
	Rectosigmoid	55 (11.0)
	Rectum	224 (45.0)
Site of colon tumor, n(%)	Right	93 (18.7)
	Left	126 (25.3)
Surgery, n(%)	Elective	479 (96.2)
	Urgent	19 (3.8)
Grade, n(%)	1	138 (27.7)
	2	172 (34.5)
	3	24 (4.8)
Lymphovascular invasion, n(%)	Yes	116 (23.3)
	No	271 (54.4)
Perineural invasion, n(%)	Yes	99 (19.9)
	No	286 (57.4)
Median examined lymph nodes (range)		14 (0-86)
Median positive lymph nodes (range)		0 (0-19)
Tumor (T), n(%)	1	10 (2.0)
	2	64 (12.9)
	3	303 (60.8)
	4	121 (24.3)
Lymph nodes (N), n(%)	N1	122 (24.5)
	N2	76 (15.3)
Stage. n(%)	I	63 (12.7)
	II	237 (47.6)
	III	198 (39.8)
Neoadjuvant CT/CRT, n(%)	Yes	64 (12.9)
	No	434 (87.1)
Adjuvant CT/CRT, n(%)	Yes	381 (76.5)
	No	117 (23.5)
Recurrence, n(%)	Yes	167 (33.5)
	No	331 (66.5)
Last Visit, n(%)	Alive	252 (50.6)
	Exitus	246 (49.4)

Kaplan-Meier analysis

**Figure 1:**
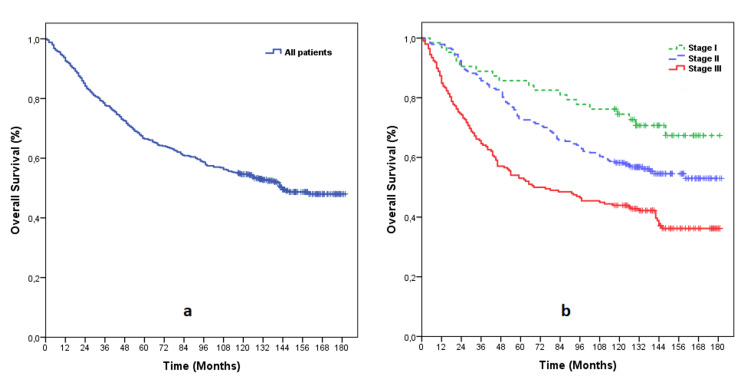
Kaplan-Meier survival curve. a. Overall survival for all patients; b. Overall survival according to stages I, II, and III.

The median time to follow-up was 123.0 (1.0-182.0) months. The median overall survival for all patients was 144.0 months (95%CI: 8.0-160.0 months) and was statistically different between the different stages (p<0.0001) (Figure 1). While the median overall survival was not reached in stage I and stage II, it was 68 months (95%CI: 32.0-104.0 months) in stage III.

The survival rates at 1, 2, 3, 5, and 10 years for all patients were 92.6%, 84.1%, 77.7%, 66.5%, and 54.5%, respectively (Table 2). The survival rates for stage I, II, and III are shown in Table 2.

**Table 2:** Survival rate of the patients according to years.

Survival rate	1-year	2-year	3-year	5-year	10-year
All patients, (%)	92.6%	84.1%	77.7%	66.5%	54.5%
Stage I, (%)	96.8%	90.5%	88.0%	85.0%	73.3%
Stage II, (%)	95.1%	89.9%	85.7%	72.6%	58.0%
Stage III, (%)	84.8%	74.2%	64.6%	52.9%	43.7%

Results of ML methods

We used ANOVA to rank the features in order of importance before applying the ML models for all periods. These features were selected on the basis of their known contribution to survival, as established in the literature. We decided to include all ranked features in the model to ensure a comprehensive analysis. According to the feature importance scores using the ANOVA algorithm, the features that are generally highly dependent on the survival variable are recurrence, lymph nodes (N), positive lymph nodes, stage, response to adjuvant treatment, perineural invasion, lymphovascular invasion and grade, which are very similar for all periods. As an example, the results of the ANOVA applied to 5-year survival are shown in Table 3.

**Table 3:** Feature importance scores for 5-year survival using ANOVA algorithm.

Feature name	F-score
Recurrence	191.19
Lymph nodes (N)	19.10
Stage	17.35
Positive lymph nodes	15.25
Tumor (T)	10.67
Response to adjuvant treatment	10.50
Perineural invasion	8.74
Adjuvant chemoradiotherapy	8.05
Lymphovascular invasion	6.50
Grade	5.18
Age	5.10
Adjuvant regimen	2.95
Adjuvant chemotherapy	2.60
Tumor localization	1.71
Smoking	1.54
Emergency surgery	1.42
Neoadjuvant chemotherapy	1.341
Neoadjuvant chemoradiotherapy	1.31
Gender	0.59
Examined lymph nodes	0.12

Twenty features from 498 patients were analyzed. DT, SVM, KNN, Ensemble Classifiers, and NN classification methods were used. The machine learning method with the highest accuracy was selected, and its score was listed with its model name. The AUC of the DT model was 0.89, which was higher than the AUC of the other models (SVM AUC: 0.84; Ensemble AUC: 0.87; NN AUC: 0.70; KNN AUC: 0.80) for predicting 1-year survival. The AUC values of the ensemble model for predicting 2-year, 3-year, and 5-year survival were 0.86, 0.92, and 0.89, respectively, which were higher than those of the other models (DT AUC: 0.82, 0.84, and 0.87; SVM AUC: 0.85, 0.88, and 0.88; NN AUC: 0.74, 0.80, and 0.79; KNN AUC: 0.83, 0.81, and 0.82). The AUC of the SVM model was 0.84, which was higher than the AUC of the other models (Decision Tree AUC: 0.81; Ensemble AUC: 0.83; NN AUC: 0.76; KNN AUC: 0.76) for predicting 10-year survival. The time-dependent F-measure and accuracy of each model over time are shown in Table 4.

**Table 4:** Performance measures of the ML models for predicting of colorectal cancer survival. F-measure represents a harmonic mean of precision, [(P) positive predictive value in ML], and recall [(R) sensitivity in ML] and is calculated as 2PR/(P+R). Accuracy (%): [(True Positive+True negative)/( True Positive+True negative+False Positive+False negative)] x 100.

		All stages		Stage I		Stage II		Stage III	
		F-measure	Accuracy (%)	F-measure	Accuracy (%)	F-measure	Accuracy (%)	F-measure	Accuracy (%)
1-year	Tree (coarse)	0.96	93.37	0.98	96.00	0.98	97.89	0.90	82.82
	SVM (linear SVM)	0.96	92.57	0.98	96.00	0.98	97.89	0.92	85.85
	Ensemble (bagged trees)	0.96	93.17	0.98	96.00	0.98	97.89	0.90	83.33
	NN (medium neural network)	0.94	89.96	0.96	93.65	0.98	97.04	0.90	83.33
	KNN (medium KNN)	0.96	93.37	0.98	96.82	0.98	97.89	0.93	87.87
2-year	Tree (coarse)	0.90	83.13	0.95	92.06	0.93	88.18	0.88	82.82
	SVM (linear SVM)	0.92	85.94	0.96	93.65	0.95	90.71	0.86	78.28
	Ensemble (boosted trees)	0.90	83.13	0.95	90.47	0.93	87.34	0.83	77.27
	NN (wide neural network)	0.90	83.13	0.95	92.06	0.92	85.65	0.85	78.28
	KNN (weighted KNN)	0.92	87.14	0.94	88.88	0.95	91.56	0.88	81.31
3-year	Tree (fine tree)	0.90	85.14	0.94	90.47	0.90	82.70	0.84	80.80
	SVM (linear SVM)	0.89	84.73	0.94	90.47	0.93	89.03	0.85	82.32
	Ensemble (bagged trees)	0.90	85.14	0.94	88.88	0.91	85.23	0.85	81.31
	NN (medium neural network)	0.89	82.93	0.93	88.88	0.92	87.34	0.82	76.76
	KNN (cosine KNN)	0.88	82.32	0.94	88.88	0.90	83.96	0.81	75.75
5-year	Tree (coarse)	0.89	86.54	0.94	90.47	0.90	86.076	0.84	84.34
	SVM (linear SVM)	0.90	87.75	0.91	85.71	0.91	87.34	0.86	85.85
	Ensemble (boosted trees)	0.90	87.14	0.94	90.47	0.90	85.23	0.86	85.85
	NN (bilayered neural network)	0.84	79.16	0.91	85.71	0.85	78.90	0.76	74.24
	KNN (medium KNN)	0.85	80.92	0.92	85.71	0.86	78.90	0.75	73.23
10-year	Tree (coarse)	0.82	79.71	0.76	68.25	0.76	73.41	0.81	82.82
	SVM (medium Gaussian SVM)	0.82	80.12	0.81	73.01	0.80	75.52	0.84	86.36
	Ensemble (boosted trees)	0.82	80.52	0.76	68.25	0.76	73.41	0.75	78.78
	NN (wide neural network)	0.73	72.08	0.80	73.01	0.71	68.77	0.72	76.76
	KNN (coarse KNN)	0.74	74.09	0.80	69.0	0.77	67.51	0.76	77.77

## Discussion

Recent studies indicate improved survival rates for patients with CRC. While advancements in screening programs and treatment methods have contributed to better outcomes over time, survival still declines as the disease progresses to more advanced stages [3]. 5-year survival rates for CRC patients are reported to be around 60.0%-65.6% [21]. In our study, the median overall survival of all patients was detected at 144 months (95%CI: 8.0-160.0 months). The median overall survival was not reached for stage I and stage II patients but was 68 months (95% CI: 32.0-104.0 months) for stage III. The survival rate for 5 years was 66.5% for all patients, similar to previous studies. The 5-year survival rates were 85.0% for stage I, 72.6% for stage II, and 52.9% for stage III.

We tested the performance of an ML model to predict the overall survival probability of CRC patients at different time intervals. The predictive ability of the models for predicting 1, 2, 3, 5, and 10-year overall survival ranged from 0.70 to 0.92 for all stages. When looking at time intervals (even over a long period such as 10 years) and considering stages separately, the accuracy of all five models was generally similar, ranging around 70.0% or higher.

In studies aiming to utilize artificial intelligence for prognosis prediction based on clinicopathological data, similar to our current retrospective study, Kuwayama et al. applied AI techniques to blood data from real-world clinical practice to evaluate the prognosis of gastric cancer patients who had undergone surgical treatment [22]. They were conducted using four ML methods, namely logistic regression (LR), random forest (RF), gradient boosting (GB), and deep neural network (DNN), to predict 5‑year survival based on clinicopathological data. The predictive accuracy and AUC were 76.8% and 0.702 for LR, 72.5% and 0.721 for RF, 75.3% and 0.73 for GB, and 76.9% and 0.682 for DNN, respectively.

The light gradient boosting machine (LGBM), which is an ensemble of multiple DTs that learn from each other to generate a more accurate final model, was used to test the performance of survival prediction in hepatocellular carcinoma (HCC) [23]. A total of 100 HCC patients were included, with a median overall survival of 43 months (range: 0.7-256 months). Although this study was conducted with a small number of patients with different types of cancer and with different ML models, it evaluated survival at different time periods, similar to our study. The survival rates at 6, 12, 24, 36, 60 and 120 months were 88%, 81%, 67%, 60%, 40% and 11%, respectively. The mean AUC of the model prediction was 0.92 for >6 months, 0.81 for >1 year, 0.78 for >2 years, 0.81 for >3 years, 0.82 for >5 years, 0.81 for >8 years, and 0.66 for >10 years.

A retrospective analysis by Alinia et al. was performed on a cohort of 284 patients with CRC who underwent surgical resection [24]. They evaluated several predictive models, including DTs, RF, random survival forests (RSF), gradient boosting, mboost, DL NN, and cox regression. Their study groups consisted of demographic and clinical data based on the hospital population, including gender, disease stage, age at diagnosis, recurrence status, and treatment details. For predicting mortality, the mboost model showed an overall accuracy of 89%. Similarly, the GB model performed well in predicting recurrence, achieving a high accuracy of 96.4%. RF showed poor overall accuracy (50%). On the other hand, DLNN had the lowest performance metrics for predicting mortality, with a sensitivity of 24%, specificity of 75%, and a low positive predictive value of 42%. Their results highlight the effectiveness of the mboost and GB models in predicting mortality in CRC patients [24]. However, in their study, ML was used to determine whether it led only to death or to relapse. Longer-term survival rates were not evaluated in this trial.

A study using data from a hospital population in South Africa evaluated six ML algorithms (logistic regression: LR, naive Bayes: NB, C5.0, RF, SVM, and artificial NN) in patients with CRC [25]. The study included patients of all stages, although the stage of 11% of the patients could not be clearly determined. Among the patients, 397 (56.9%) were non-metastatic. Notably, the study did not provide specific details about the localization of the cancer. The ANN achieved the highest AUC and accuracy for predicting cancer recurrence (87.0% AUC and 81.0% accuracy) and patient survival (82.0% AUC and 77.0% accuracy). Other models demonstrated comparable performance to ANN. Notable risk factors for CRC recurrence included radiological stage, patient age, histology, and race, each of which contributed differently to patient survival.

In another study, ML models were applied to an Australian CRC dataset to predict short-term (within 1 year) and long-term (within 5 years) survival of CRC patients [26]. Among the cases, 779 (63.0%) had tumors located in the colon, while 457 (37%) had tumors in the rectum. In the study, 87.3% (n=1079) of the patients were classified as M0 and 12.7% (n=157) as M1. They used 11 ML algorithms: LR, DT classifier, random forest (RF) classifier, K-neighbors classifier, Gaussian naive Bayes, multinomial naive Bayes, C-support vector classifier, stochastic gradient descent classifier, GB classifier, LGBM classifier, and extreme gradient boost classifier. The best-performing model was logistic regression with an AUC of 0.850 for the 1-year and 0.872 for the 5-year survival prediction. Accuracy rates were reported as 0.90 for 1 year, 0.86 for 2 years, 0.83 for 3 years, 0.83 for 4 years, and 0.82 for 5 years. However, this study did not provide data on survival prediction beyond 5 years, although it included a large number of patients and used more algorithms. In addition, while stages T and N were generally provided, details of stages I-II-III were not available, and survival estimates by years for metastatic and non-metastatic groups were not included.

When reviewing studies designed according to the CRC stage, there is no study specifically designed to use AI models to predict survival based on clinical data. Researchers studied 259 stage II patients and developed a novel predictive model using a retrospective clinical database [27]. This model aimed to predict recurrent high-risk stage II CRC with high accuracy using auto-AI prediction one software. The study evaluated the importance of variables using a permutation feature importance method to assess DFS. The AUC for the AI model was 0.775. Notably, preoperative carcinoembryonic antigen levels >5.0 ng/mL, venous invasion, and obstruction were identified as high-risk factors contributing to cancer recurrence. Patients with ≥2 of these factors are considered to be at high risk of recurrent stage II CC and may benefit from adjuvant chemotherapy.

There are still insufficient studies using AI with clinical data to predict cancer prognosis. To the best of our knowledge, this study is the first to evaluate different ML models for predicting both overall survival and long-term survival at different time points in non-metastatic (stage I to III) CRC using hospital population data, which will contribute to the literature and inspire further studies.

The main limitation of our study was its retrospective nature, which was limited by the available clinical variables and potential biases arising from data collection practices. Larger studies in different geographical regions and including individuals from different ethnic backgrounds will be essential to provide high-quality evidence for clinical application. Although these ML models are widely used to predict survival, each has specific limitations. For example, DTs often face challenges with overfitting, while SVMs can be computationally burdensome for large datasets. The limitation of KNN is computational complexity, which increases with dataset the size of the dataset, while ensemble models increase model complexity by integrating multiple models. Neural networks require significant computational resources due to their reliance on large data sets and complex model structures.

## Conclusions

There is no optimal model in the literature for predicting cancer prognosis, and most of the developed models that have been developed still need to be validated. However, we hope that with the increase in studies based on readily available clinical data, such as our study, our current knowledge will increase, and we will be able to benefit our patients by integrating the data obtained into clinical practice.
